# Lipid peroxidation-induced ferroptosis as a therapeutic target for mitigating neuronal injury and inflammation in sepsis-associated encephalopathy: insights into the hippocampal PEBP-1/15-LOX/GPX4 pathway

**DOI:** 10.1186/s12944-024-02116-x

**Published:** 2024-04-29

**Authors:** Haosen Wang, Lixiao Xu, Xiaojuan Tang, Zhen Jiang, Xing Feng

**Affiliations:** 1 Department of Neonatology, Children’s Hospital of Soochow University, Suzhou, 215003 Jiangsu China; 2 Department of Critical Care Medicine, Xuzhou Children’s Hospital, Xuzhou, 221002 Jiangsu China

**Keywords:** Sepsis-associated encephalopathy, Lipid peroxidation, Ferroptosis, PEBP-1/15-LOX/GPX4

## Abstract

**Background:**

Sepsis-associated encephalopathy (SAE) refers to the widespread impairment of brain function caused by noncentral nervous system infection mediated by sepsis. Lipid peroxidation-induced ferroptosis contributes to the occurrence and course of SAE. This study aimed to investigate the relationship between neuronal injury and lipid peroxidation-induced ferroptosis in SAE.

**Methods:**

Baseline data were collected from pediatric patients upon admission, and the expression levels of various markers related to lipid peroxidation and ferroptosis were monitored in the serum and peripheral blood mononuclear cells (PBMCs) of patients with SAE as well as SAE model mice. The hippocampal phosphatidylethanolamine-binding protein (PEBP)-1/15-lysine oxidase (LOX)/ glutathione peroxidase 4 (GPX4) pathway was assessed for its role on the inhibitory effect of ferroptosis in SAE treatment.

**Results:**

The results showed elevated levels of S100 calcium-binding protein beta (S-100β), glial fibrillary acidic protein, and malondialdehyde in the serum of SAE patients, while superoxide dismutase levels were reduced. Furthermore, analysis of PBMCs revealed increased transcription levels of *PEBP1*, *LOX*, and long-chain fatty acyl-CoA synthetase family member 4 (*ACSL4*) in SAE patients, while the transcription levels of *GPX4* and cystine/glutamate transporter xCT (*SLC7A11*) were decreased. In comparison to the control group, the SAE mice exhibited increased expression of S-100β and neuron-specific enolase (NSE) in the hippocampus, whereas the expression of S-100β and NSE were reduced in deferoxamine (DFO) mice. Additionally, iron accumulation was observed in the hippocampus of SAE mice, while the iron ion levels were reduced in the DFO mice. Inhibition of ferroptosis alleviated the mitochondrial damage (as assessed by transmission electron microscopy, hippocampal mitochondrial ATP detection, and the JC-1 polymer-to-monomer ratio in the hippocampus) and the oxidative stress response induced by SAE as well as attenuated neuroinflammatory reactions. Further investigations revealed that the mechanism underlying the inhibitory effect of ferroptosis in SAE treatment is associated with the hippocampal PEBP-1/15-LOX/GPX4 pathway.

**Conclusion:**

These results offer potential therapeutic targets for the management of neuronal injury in SAE and valuable insights into the potential mechanisms of ferroptosis in neurological disorders.

**Supplementary Information:**

The online version contains supplementary material available at 10.1186/s12944-024-02116-x.

## Background

Sepsis-associated encephalopathy (SAE) refers to the widespread impairment of brain function caused by sepsis that is mediated by noncentral nervous system infections. Statistical data show that SAE is observed in approximately 50% of sepsis patients [1, 2]. During the acute phase, SAE patients exhibit consciousness disorders and an increased mortality rate [3]. Survivors may experience long-term sequelae, including cognitive impairment, decreased quality of life, and behavioral changes [4]. The exact cause of SAE is unknown, but it is believed to be associated with endothelial cell dysfunction, disruption of the blood–brain barrier, abnormal cerebral perfusion, and abnormal neurotransmitter release [5].

The oxidative reaction of lipid molecules in cells brought on by reactive oxygen species is known as lipid peroxidation, and it alters the composition and functionality of cell membranes [6]. A unique type of cell death called ferroptosis is caused by the dysregulation of intracellular iron ions [7]. Its mechanisms include iron accumulation in cells, reactive oxygen species generation, and disruption of DNA repair pathways [8]. According to studies, lipid peroxidation can cause ferroptosis during neuronal injury, further aggravating brain injury. Therefore, lipid peroxidation and ferroptosis play important roles in neuronal injury [9]. In addition, lipid oxidation damage resulting from lipid peroxidation leads to abnormal cell function and inflammatory responses, thereby exacerbating the severity of neuronal injury [10]. Ferroptosis, a recently identified form of cell death, remains incompletely understood in terms of its specific mechanisms. However, research indicates that ferroptosis plays a significant role in the progression of neurological injuries. During this process, the accumulation of intracellular iron ions and the development of oxidative stress result in membrane rupture and cell death. Further understanding of the underlying mechanisms of ferroptosis in the context of neurological injuries will contribute to uncovering its role in pathological processes and provide novel insights for developing therapeutic strategies for neurological injuries.

This study aimed to explore the molecular mechanism of lipid peroxidation-induced ferroptosis in SAE-related neuronal injury. A SAE mouse model was established using cecal ligation and puncture (CLP) [11], and the changes in hippocampal cell damage-related mechanisms and biomarkers, such as phosphatidylethanolamine-binding protein 1 (PEBP-1), 15-lysyl oxidase (15-LOX), and glutathione peroxidase 4 (GPX4) [12], in SAE mice were studied by inhibiting ferroptosis with deferoxamine (DFO) [13]. PEBP-1 is an important intracellular regulatory protein that regulates cellular processes such as apoptosis, oxidative stress, and inflammation. 15-LOX is a crucial target molecule regulated by PEBP-1. 15-LOX catalyzes lipid oxidation reactions, leading to the production of biologically active metabolites. GPX4 is also an important effector molecule regulated by PEBP-1. In addition, GPX4 is a significant antioxidant enzyme that eliminates peroxides within cells, maintaining the cellular redox balance. The relationship between SAE and the PEBP-1/15-LOX/GPX4 signaling pathway is still unclear. By studying the relationship between lipid peroxidation and ferroptosis, new insights and methods for the treatment of SAE-related neuronal injury can be developed, contributing to the recovery of SAE patients.

## Methods

### Mouse model of SAE and grouping

A total of 81 mice (C57BL/6 J male, 6–8 weeks old, 20–25 g) were obtained from the Animal Experiment Center of Xuzhou Medical University. The ethical approval code for the animal experiments is 2021–12-W023. The mice (with unrestricted access to food and water) were kept in a setting with the temperature ranging from 22 °C to 26 °C and a humidity between 40 and 70%. They were randomly divided into three groups: sham, SAE, and DFO. Based on the duration of the experiment, each group was divided into three subgroups: 1 day, 3 days, and 7 days. The SAE and DFO groups were established using the CLP method [11]. Specifically, the mice underwent a 12-h fast (food and water) prior to the operation, then they were anesthetized via an intraperitoneal injection of ketamine (100 mg/kg) and xylazine (10 mg/kg), followed by a 1-cm incision along the midline of the abdomen to expose the cecum. A silk thread was used to bind the midpoint between the ileocecal valve and the end of the cecum, after which a 21G needle was used to puncture the distal cecum and squeeze out a little amount of excrement. The cecum was restored into the abdominal cavity, and the peritoneum and skin were sutured. In the sham group, the cecum was exposed after the laparotomy, without ligation or puncture. After surgery, all mice were exposed to an intraperitoneal injection of preheated normal saline (37 °C, 50 mL/kg). The DFO group received three intraperitoneal injections of DFO (50 mg/kg) at 12 h before, immediately after, and 12 h after the operation, while the other groups received intraperitoneal injections with normal saline (same volume). The animals were observed every 12 h after the administration and operation. The method used for euthanizing and collecting specimens from the experimental animals was the same as that used before modeling with an intraperitoneal injection of ketamine (100 mg/kg) and xylazine (10 mg/kg).

### Clinical sample collection

Ten children admitted to the Pediatric Intensive Care Unit of Xuzhou Children’s Hospital from September 2022 to August 2023 with SAE were randomly selected as the study group (the SAE group), and 10 sepsis patients without associated brain dysfunction were selected as the control group (the non-SAE group). This study was approved by the Medical Ethics Committee of Xuzhou Children’s Hospital (Approval No. 2022–05-07-H07). The criteria for inclusion in the SAE group were as follows: (1) diagnosis of sepsis and organ dysfunction based on the 2020 version of the Surviving Sepsis Campaign International Guidelines for the Management of Septic Shock and Sepsis-Associated Organ Dysfunction in Children [14]; (2) age of > 28 days and ≤ 14 years; (3) presence of an altered mental status upon admission, such as seizures, delirium, or coma, with a Glasgow Coma Scale score ≤ 9; and (4) abnormalities observed in the electroencephalogram and cranial imaging. The exclusion criteria were as follows: (1) underlying neurological disorders affecting the nervous system; (2) diagnosis of intracranial infection, intracranial hemorrhage, or other intracranial organic lesions during hospitalization; and (3) brain disorders caused by dysfunction of other organ systems, such as hepatogenic encephalopathy, renal encephalopathy, etc. The baseline data, Glasgow Coma Scale scores, and white blood cell count, C-reactive protein, alanine aminotransferase, aspartate aminotransferase, blood urea nitrogen, serum creatinine, and blood culture results from the two groups of hospitalized pediatric patients were collected. Blood samples from patients were obtained at 24, 48, and 72 h following admission, and the serum and peripheral blood mononuclear cells (PBMCs) were separated. The levels of S100 calcium-binding protein beta (S-100β), glial fibrillary acidic protein (GFAP), superoxide dismutase (SOD), and malondialdehyde (MDA) at each time point were detected in the serum by using enzyme-linked immunosorbent assays (ELISAs). The ELISA kits were purchased from Shanghai Lanpai Biological Technology Co., Ltd. The extracted PBMCs were preserved in 1 mL of TRIzol reagent (Vazyme Biotech, Nanjing, China) and stored in a -80 °C freezer.

### Total RNA extraction and real-time polymerase chain reaction (qPCR)

PBMCs were extracted using the procedure for lymphocyte separation. The procedure consisted of collecting the patient’s blood specimen (usually 2 mL) and centrifuging it at 2000 rpm for 5 min to separate the blood cells. Next, 4 mL of PBS buffer was added to the separated blood cells, and the mixture was mixed well. Subsequently, 2 mL of Ficoll reagent (New England Biolabs Inc., Beijing, China) was slowly added, and the mixture was allowed to form two layers, with the lower layer being a liquid. The two layers underwent centrifugation at 1800 rpm for 30 min. The solution separated into three layers, with the PBMC layer in the middle appearing as a cloudy layer. The PBMC layer in the middle was carefully removed, PBS buffer was added to give a total volume of 10 mL, and it was centrifuged at 2000 rpm for 5 min. The PBMCs adhered to the bottom of the centrifuge tube, so they were washed twice with PBS, 1 mL of TRIzol was added, and the tube was sealed and stored at -80 °C for subsequent qPCR experiments. The total RNA isolated from the PBMCs of the patients was reverse transcribed to complementary DNA by a PrimeScript™ RT reagent kit (Vazyme Biotech, Nanjing, China) and stored at –20℃. The qPCR was carried out to detect the mRNA levels. Using β-actin as the internal reference, the cDNA was amplified using SYBR Green Mix (Biosharp, Hefei, China) on a LightCycler 96 fluorescence qPCR system. The PCR conditions were as follows: 1 min of incubation at 94 °C, then 35 cycles of denaturation at 94 °C for 30 s, annealing at 55 °C for 30 s, and extension at 72 °C for 30 s. The relative expression level of the target gene was calculated using the 2^−ΔΔCt^ method. Table 1 lists the primer sequences used to amplify the target genes.

**Table 1:** Primer sequences for qPCR

Gene		Primer sequence (5′ to 3′)
*GPX4*	Forward	GAGGCAAGACCGAAGTAAACTAC
	Reverse	CCGAACTGGTTACACGGGAA
*PEBP1*	Forward	CCTGCAAGAAGTGGACGAG
	Reverse	ACCAAGGTGTAGAGCTTCCCT
*12-LOX*	Forward	ATGGCCCTCAAACGTGTTTAC
	Reverse	GCACTGGCGAACCTTCTCA
*15-LOX*	Forward	GGGCAAGGAGACAGAACTCAA
	Reverse	CAGCGGTAACAAGGGAACCT
*β-actin*	Forward	GCACAGAGCCTCGCCTT
	Reverse	GTTGTCGACGACGAGCG
*SLC7A11*	Forward	TCTCCAAAGGAGGTTACCTGC
	Reverse	AGACTCCCCTCAGTAAAGTGAC
*ACSL4*	Forward	CATCCCTGGAGCAGATACTCT
	Reverse	TCACTTAGGATTTCCCTGGTCC

### Neuroethological score

The neurobehavioral changes in the different groups at 1-day postoperation were evaluated by the neuroethological scores [15], according to the following procedures and criteria: (1) The corneal reflex was tested by gently touching the mouse’s cornea with a cotton swab to elicit blinking or shaking the head. (2) The auricular reflex was considered normal if the mouse’s auricle was touched and caused a strong head rotation. (3) The righting reflex was normal if the mouse was placed in the supine position and quickly returned to the prone position, flattening the front and rear feet. (4) The Tai-flick reflex was normal if the mouse turned around and escaped injury after the tail was briefly stimulated. (5) The escape reflex was normal if the mouse received a temporary stimulus and escaped. The scoring criteria were as follows: normal reflex (within 1 s), 2 points; reflex dullness (1–10 s), 1 point; and areflexia, 0 points. The total score was calculated by summing each item, with a scale of 1 to 10.

### Hematoxylin and eosin (H&E) staining and transmission electron microscopy (TEM) detection

One day after the operation, three mice from each group were sacrificed, and their brains were removed. The hippocampi of the left brains were isolated and immediately immersed in a 4% paraformaldehyde universal tissue fixative (Biosharp, Hefei, China). The tissue embedding, tissue sectioning, and H&E staining were performed before the section scanning. The right brains of the mice were also removed, immediately immersed in a TEM fixative (Servicebio, Wuhan, China), and stored at 4 °C. After 30 min, the samples were taken out, and the hippocampi were immediately placed in an ice bath. Tissue samples (2 mm × 2 mm) were prepared and immersed in the TEM fixative, followed by storage at 4 °C. Finally, the tissues were analyzed by TEM.

### Protein extraction and western blot

First, the tissue sample was rapidly frozen in liquid nitrogen to maintain its integrity and stability. Next, the frozen tissue sample was placed into a prechilled homogenization tube. Then, a homogenizer was used to break down the sample. Radioimmunoprecipitation assay lysate (Beyotime Biotechnology, Shanghai, China) and phenylmethylsulfonyl fluoride (Beyotime Biotechnology, Shanghai, China) were mixed in a ratio of 50 to 1 to extract the total protein in the hippocampal tissues (*n* = 3 in each group). The protein concentration of the extract was determined by a bicinchoninic acid kit (Beyotime Biotechnology, Shanghai, China), and then all samples were diluted to the same protein concentration. A sodium dodecyl sulfate–polyacrylamide gel electrophoresis rapid preparation kit (Beyotime Biotechnology, Shanghai, China) was used to prepare a 10–12% gelatinous plate, and the same amount of tissue protein (20 µg) in each group was added to each well. After gel electrophoresis and transfer, the polyvinylidene fluoride (PVDF) membranes were washed and blocked with 5% skim milk powder for 1 h before incubation with primary antibodies against GPX4 (Abcam, Cambridge, UK, ab125066, 1:1000), PEBP-1 (Abcam, Cambridge, UK, ab76582, 1:1000), 15-LOX (Proteintech, Chicago, IL, USA, 13073–1-AP, 1:500), and GAPDH (Affinity, Changzhou, China, AF7021, 1:10,000) at 4 °C overnight. After washing, the PVDF membranes were treated with horseradish peroxidase-coupled antibody (Bioworld, Shanghai, China, BS13278, 1:10,000) at room temperature for 1 h. Pierce ECL GAPDH served as the internal control for total protein when detecting the protein bands using Chemiluminescent Substrate (Biosharp, Hefei, China).

### ELISA

The levels of S-100β, neuron-specific enolase (NSE), and 4-hydroxynonenal (4-HNE) in the serum as well as interleukin-6 (IL-6), tumor necrosis factor alpha (TNF-α), and MDA in the hippocampal tissues of the mice were measured using commercial ELISA kits (Lanpaibio, Shanghai, China) (*n* = 4 for each group). The blood was obtained by removing the mouse eyeballs, followed by centrifugation to separate the serum. The hippocampus was obtained and homogenized in normal saline. After centrifugation, the protein concentrations were measured, and the samples were subsequently diluted to the same protein concentration. The levels of S-100β, MDA, total SOD, and GFAP in the serum of patients were measured using commercial ELISA kits (Lanpaibio, Shanghai, China).

Fifty microliters of a standard working solution at various concentrations was applied to each well in a 96-well plate. Next, the samples were added to the tested wells, with 10 μL in each well, followed by 40 μL of sample diluent in each well. Next horseradish peroxidase-labeled antibody (100 μL) was added to each well and incubated for 60 min at 37 °C. After discarding the liquid, the plate was patted dry on absorbent paper, followed by each well being filled with detergent and incubation for 1 min. The plate was once more patted dry and then cleaned five times after disposing of the detergent. Each well was filled with 50 μL of substrate working solutions A and B, which were then incubated for 15 min at 37 °C in the dark. Following the addition of 50 μL of the stop-working solution to each well, the optical density (OD) value of each well at 450 nm was determined in less than 15 min using a microplate reader.

### Detection of ferrous ions by a colorimetric method

The concentration of ferrous ions was determined in hippocampal tissues (*n* = 4 per group) using a Ferrous Ion Colorimetric Kit (Elab Science, Wuhan, China). Colorimetry was used in this study to detect ferrous ions. The principle is as follows: Ferrous ions in the sample bind to a probe, resulting in the formation of a substance that exhibits a strong absorption peak at a wavelength of 593 nm. Within a certain range, the OD of this substance is linearly correlated with the concentration of ferrous ions, reflecting the total content of ferrous ions. The hippocampal samples and iron standard working solution were prepared. Next, 150 μL of the chromogenic solution was added to 300 μL of the standard working solution and the sample to be tested, and the mixture was thoroughly mixed. The mixture was incubated at 37℃ for 10 min and subsequently centrifuged at 12,000 × *g* for 10 min. Then, it was added to the enzyme-labeled plate, and the OD value was measured at 593 nm. The concentration of ferrous ions in the hippocampal tissues was calculated according to the provided instructions.

### Detection of the mitochondrial membrane potential (MMP) with JC-1

The mitochondria were extracted from the hippocampal tissues of mice (*n* = 4 per group) using a mitochondrial extraction kit (Solarbio, Beijing, China). The homogenization of hippocampal tissue was performed in an ice-cold bath at 0 °C, followed by centrifugation at 4 °C to extract the mitochondria, which were then rapidly transferred and stored at -70 °C. The proteins were measured by the bicinchoninic acid (BCA) method and then diluted to the same concentration. JC-1 staining working solution was prepared following the instructions provided in the MMP detection kit (Beyotime Biotechnology, Shanghai, China) and was subsequently mixed with the extracted hippocampal mitochondria. The preparation and addition of JC-1 working solution were also performed in a sterile ice-cold bath at 0 °C, followed by centrifugation at 4 °C. The fluorescence intensities of the JC-1 polymer and monomer were measured at 525/590 nm and 490/530 nm, respectively.

### Detection of the mitochondrial ATP content in the hippocampus

The ATP content was determined utilizing the luciferin–luciferase luminescence technique. Hippocampal mitochondria were isolated from mice (*n* = 4 per group), and the protein content was measured by a BCA protein assay reagent. The sample (50 μL) and the luciferin–luciferase reaction kit reagent (50 μL) (Beyotime Biotechnology, Shanghai, China) were reacted to quantify the ATP content. According to the instructions, ATP in the samples is unstable at room temperature but can remain stable for up to 6 h on ice (0 °C). Therefore, sample lysis, working solution preparation, and other operations were conducted under sterile conditions in an ice bath at 0 °C, and centrifugation was performed at 4 °C. Luminescence was measured using a luminometer, and the ATP content was quantified using a standard curve.

### Statistical analysis

The analysis of the data was done with SPSS Statistics 23.0. The chart was created using GraphPad Prism 8.0 software. The data were obtained from at least three independent tests and presented as the mean ± standard deviation. The t-test (two-independent-sample) was used to compare the normally distributed data of the two groups, and one-way analysis of variance was utilized. *P* < 0.05 was used as the significance criterion to assess statistical significance.

## Results

### Neuronal injury in SAE patients is associated with lipid peroxidation-induced ferroptosis

**Fig. 1:**
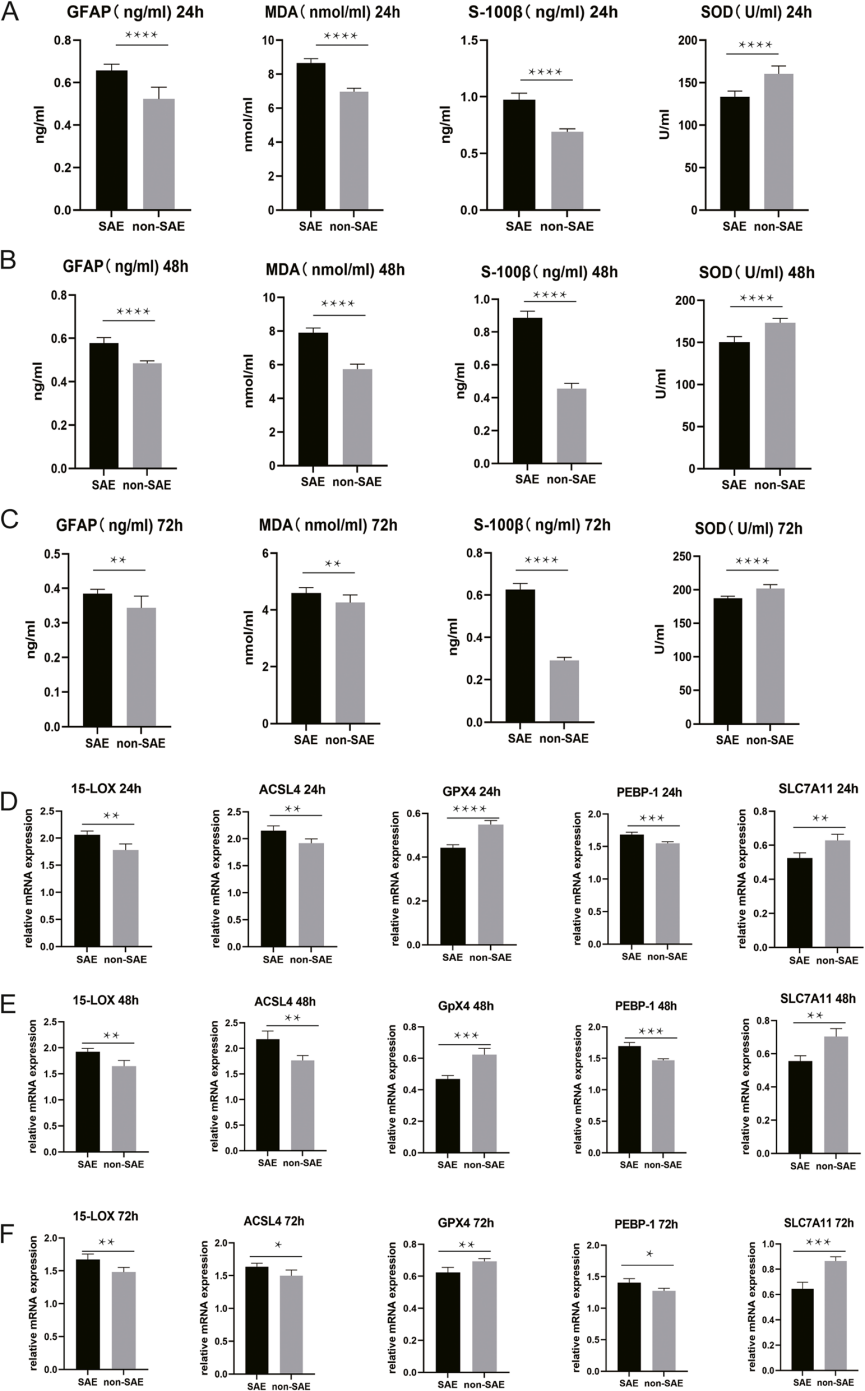
Neuronal injury in SAE patients was associated with lipid peroxidation-induced ferroptosis. **A**–**C** At 24, 48, and 72 h after admission, the whole blood was collected from the patients, and the serum was separated. The levels of S-100β, GFAP, SOD, and MDA in the serum at each time point were detected using ELISA. **D**–**F** At 24, 48, and 72 h after admission, the whole blood was collected from the patients, and the PBMCs were separated. The mRNA levels of *PEBP1*, *GPX4*, *SLC7A11*, *15-LOX*, and *ACSL4* at each time point were detected using qPCR (*N* = 10, ^*^P < 0.05, ^**^P < 0.01, ^***^P < 0.001, ^****^P < 0.0001)

For the pediatric patients in the SAE group and the non-SAE group, the baseline data of the Glasgow Coma Scale, white blood cell count, C-reactive protein, alanine aminotransferase, aspartate aminotransferase, blood urea nitrogen, serum creatinine, and blood culture results were collected (Supplementary Table 1). At 24, 48, and 72 h after admission, the whole blood was collected from the patients, and the serum and PBMCs were isolated. ELISAs were performed to detect the levels of S-100β, GFAP, SOD, and MDA in the serum. The serum levels of S-100β, GFAP, and MDA in the SAE group were significantly greater than those of the control group at 24, 48, and 72 h after admission, while the SOD level was significantly lower compared to the control group (Fig. 1A–C). Furthermore, the serum levels of S-100β, GFAP, and MDA showed significant fluctuations at 72 h. Moreover, qPCR analysis of isolated PBMCs revealed that at 24, 48, and 72 h after admission, the transcription levels of *PEBP-1*, *15-LOX*, and acyl-CoA synthetase long chain family member 4 (*ACSL4*) in the peripheral blood were significantly greater in the SAE group compared to the control group, while the transcription levels of *GPX4* and *SLC7A11* were significantly less (Fig. 1D–F). The transcription levels of *PEBP-1*, *GPX4*, *SLC7A11*, and *ACSL4* also showed significant fluctuations at 72 h.

### Neuronal injury in the SAE mice is associated with lipid peroxidation-induced ferroptosis

**Fig. 2:**
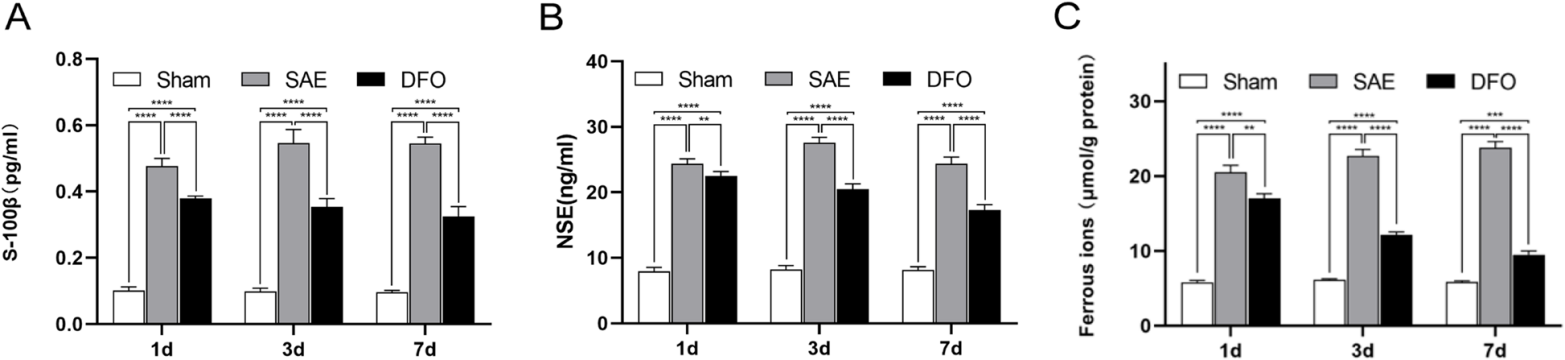
Neuronal injury in the SAE model mice was associated with lipid peroxidation-induced ferroptosis. **A**–**B** The levels of S-100β and NSE in the serum of different groups at 1, 3, and 7 days were detected by ELISA. **C** The ferrous ion concentrations in the hippocampi at the three time points were determined by chemiluminescence. (*N* = 4, ^*^P < 0.05, ^**^P < 0.01, ^***^P < 0.001, ^****^P < 0.0001)

The S-100β and NSE levels of the SAE group were greater than those of the sham group, while the expression levels of S-100β and NSE were less in the DFO group at the three time points (Fig. 2A, B). Compared to the DFO group, the ferrous ion concentration in the hippocampus was greater in the SAE group at all time points, while it was less in the sham group (Fig. 2C). These results revealed hippocampal injury after CLP, suggesting effective modeling. The brain injury induced by SAE may be caused by accumulated ferrous ions. Besides, the DFO may improve hippocampal injury and the accumulation of ferrous ions.

### Inhibiting ferroptosis alleviates SAE mitochondrial damage and oxidative stress

**Fig. 3:**
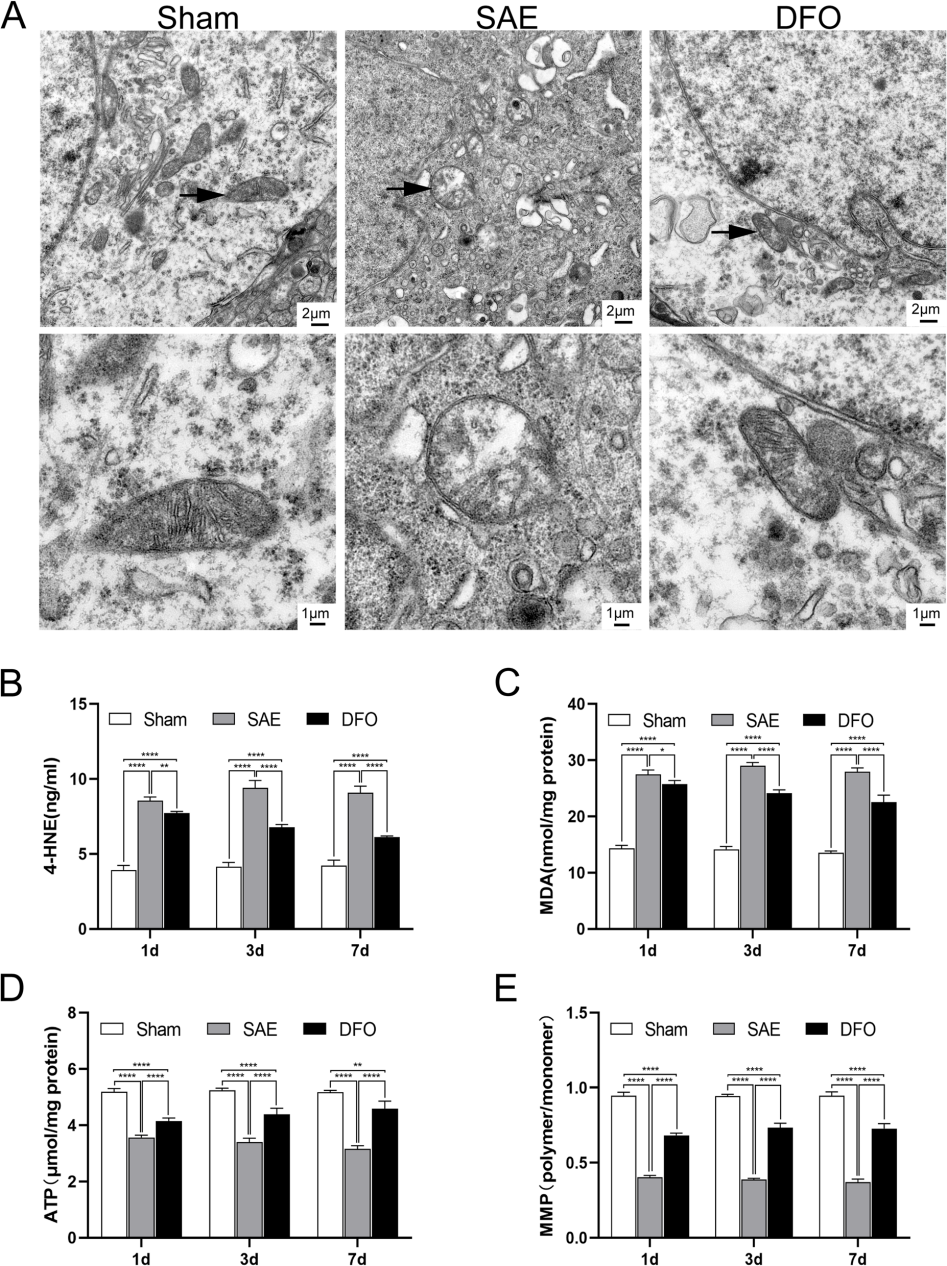
Suppressing ferroptosis alleviated SAE mitochondrial damage and oxidative stress. **A** TEM characterization of hippocampal cells showing the structural morphology and damage of the hippocampal mitochondria in different groups of mice. **B**, **C** ELISA detection of the MDA and 4-HNE levels. **D** The ATP content in the hippocampal mitochondria. **E** The ratio of MMP JC-1 polymer to monomer in the hippocampus. (*N* = 4, ^*^P < 0.05, ^**^P < 0.01, ^***^P < 0.001, ^****^P < 0.0001)

In the sham group, at 1 day after the operation, TEM showed intact mitochondria cristae in the hippocampus, with no significant abnormalities in the membrane. In contrast, the SAE group had fewer mitochondrial cristae of the hippocampus, an increased membrane density, ruptured membranes, and partial vacuolar degeneration. For the DFO group, some mitochondrial cristae had decreased, the membrane density had slightly increased, and there was no evidence of rupture or vacuolar degeneration (Fig. 3A). Additionally, the levels of the active aldehyde 4-HNE in the serum and MDA in the hippocampi of the DFO group were greater compared to the sham group, but lower compared to the SAE group (Fig. 3B, C). In addition, at the same time point, the ATP content in the hippocampal mitochondria and the MMP were less in the SAE group than in the sham group. However, the ATP content and the MMP were greater in the DFO group compared to the SAE group (Fig. 3D, E). These results revealed mitochondrial damage in SAE mice and suggested that lipid peroxidation may contribute to hippocampal injury and mitochondrial damage.

### Inhibiting ferroptosis reduces SAE neuroinflammation

**Fig. 4:**
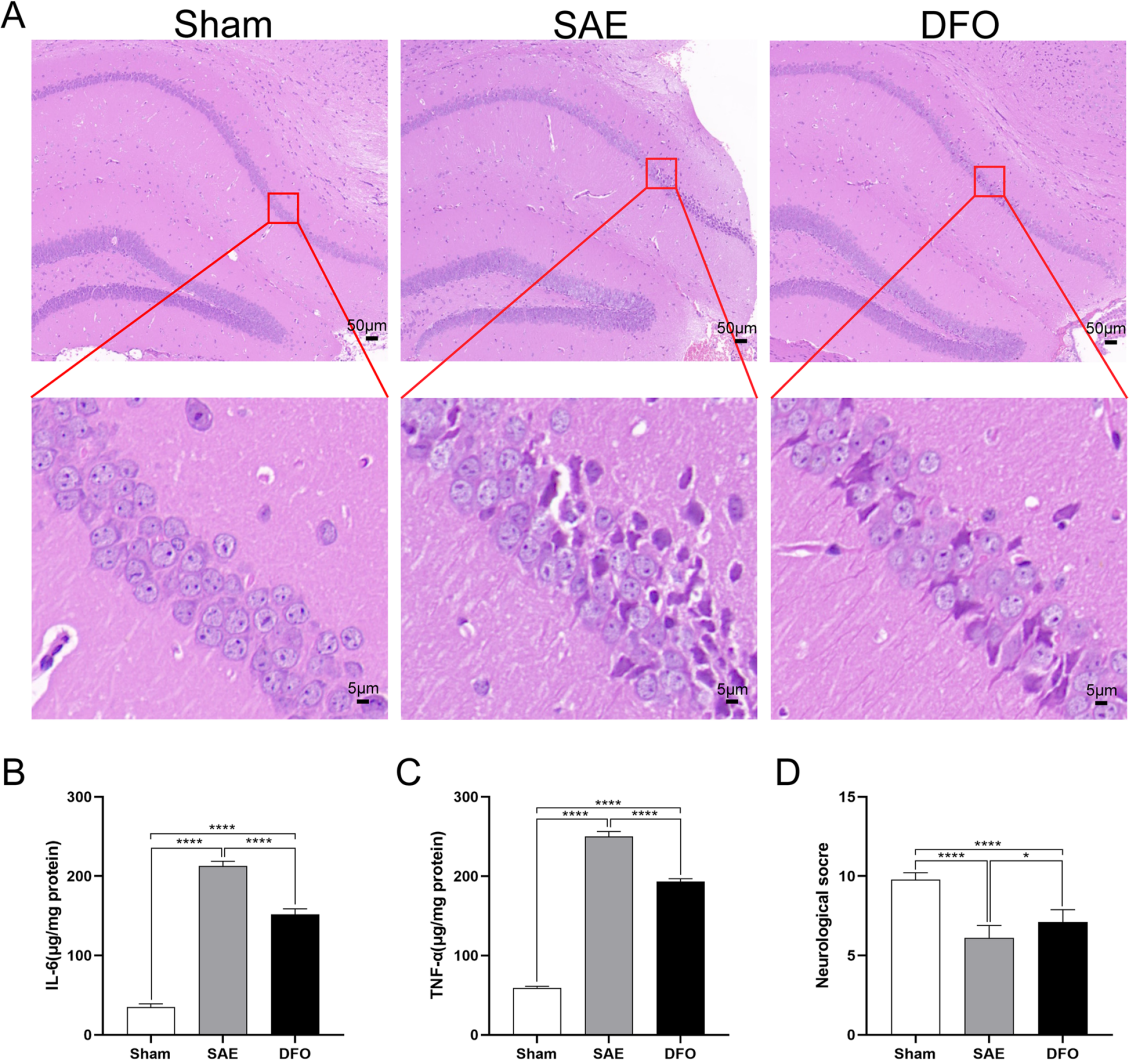
Ferroptosis inhibition reduced SAE neuroinflammation. **A** H&E staining of cells in the hippocampal CA1 zone from the different groups at 1 day after CLP. The low-magnification images (first row) mainly show the location of cell damage areas within the hippocampal structure, while the high-magnification images (second row) provide more detailed information on the arrangement and damage of cells. **B**, **C** The expression levels of IL-6 and TNF-α in the hippocampi of the different groups at 1 day after modeling were detected by ELISA. **D** Neurobehavioral changes in the different groups at 1-day post operation were evaluated by the neuroethological scores

At 1 day after model induction, H&E staining showed an orderly arrangement of cells in the hippocampal CA1 zone of the sham group. In contrast, the SAE group exhibited sparse and disordered cells, with some cells displaying structural abnormalities. The DFO group showed a regular cell arrangement, albeit with some cellular damage (Fig. 4A). The neuroethological score of the SAE mice was less than that of sham group at 1 day post CLP, but the neuroethological score of the DFO group was greater than that of the SAE group (Fig. 4D). Meanwhile, the levels of IL-6 and TNF-α of the SAE group were greater than those of the other groups at 1 day after model induction (Fig. 4B, C).

### The mechanism of ferroptosis inhibition for treating SAE neuronal injury is related to the hippocampal PEBP-1/15-LOX/GPX4 pathway

**Fig. 5:**
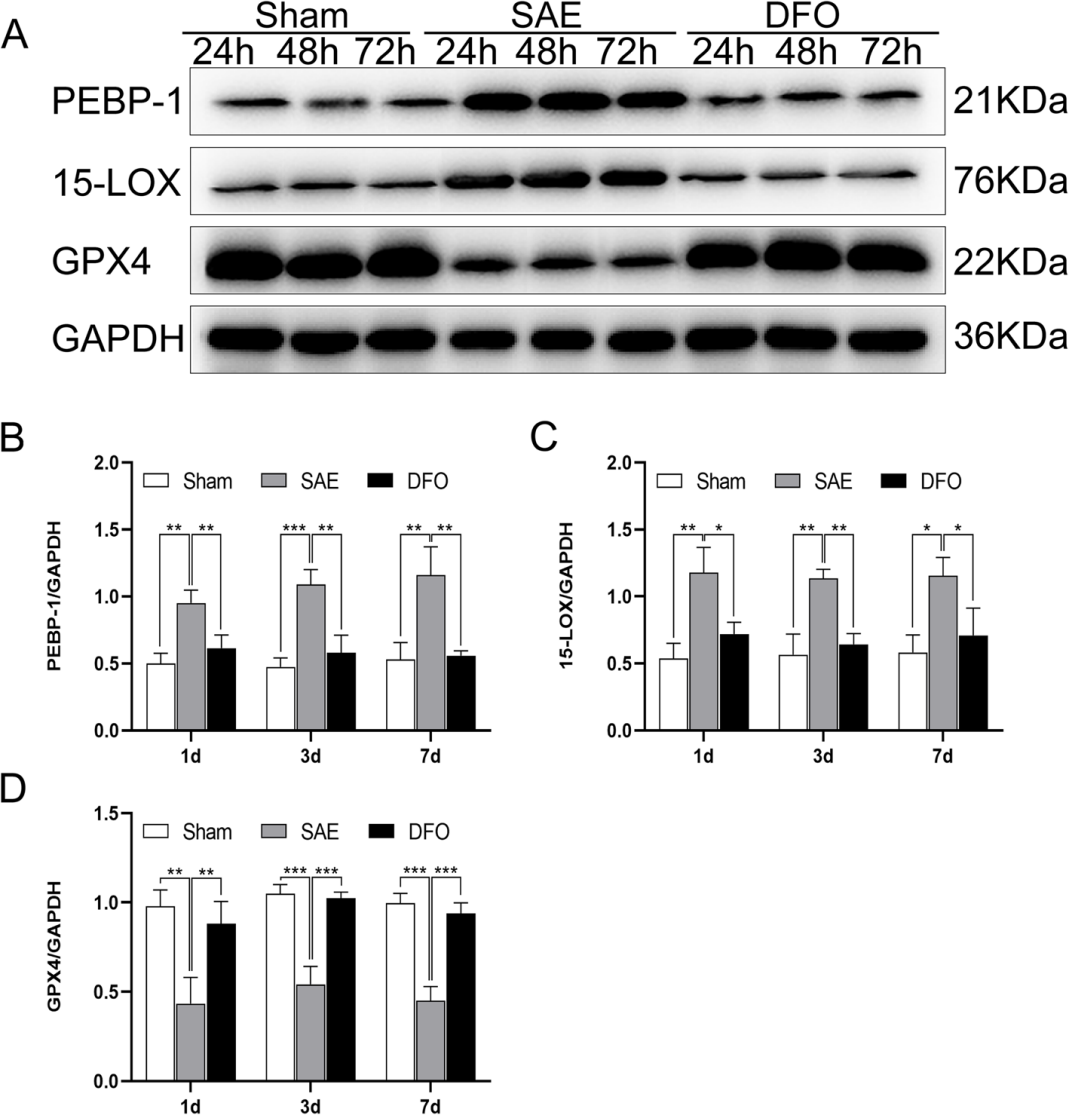
The mechanism of ferroptosis inhibition for the treatment of SAE neuronal injury was related to the hippocampal PEBP-1/15-LOX/GPX4 pathway. **A** Representative western blot images of PEBP-1, 15-LOX, GPX4 proteins in the hippocampus of the different groups at 1, 3, and 7 days (normalized to GAPDH). **B**–**D** The levels of PEBP-1, 15-LOX, and GPX4 proteins in the hippocampus of all the groups at the three time points (normalized to GAPDH). (*N* = 3, ^*^P < 0.05, ^**^P < 0.01, ^***^P < 0.001, ^****^P < 0.0001)

The western blot results revealed that the PEBP-1 and 15-LOX levels in the hippocampal tissues of the SAE group were greater than those of the sham group, while the GPX4 level in the hippocampal tissues of the SAE group was less than that of the sham group. The PEBP-1 and 15-LOX levels in the hippocampal tissues of the DFO group were less than those of the SAE group, while the GPX4 level in the hippocampal tissues of the DFO group was greater than that of the SAE group at the three time points (Fig. 5A–D). These results suggested that hippocampal injury caused by SAE might be related to the highly expressed proteins PEBP-1 and 15-LOX as well as inhibited GPX4 activity, whereas DFO could inhibit the expression of PEBP-1 and 15-LOX and restore GPX4 activity.

## Discussion

SAE is a complication commonly observed in the early stages of sepsis, with a high incidence rate [16]. Clinically, SAE is characterized by different levels of consciousness disorders in the acute stage, such as delirium, coma, convulsions, and polyneuropathy [17]. An observational study based on the claim data of the largest insurance company in Germany showed that the incidence of dementia in sepsis patients was 2–3 times greater than that in patients without sepsis after 2 years [18]. Additionally, a retrospective cohort study spanning a 14-year follow-up period reported that the risk of senile sepsis patients developing dementia, especially vascular cognitive impairment, doubled, with the highest risk occurring at 3–12 months after infection and lasting for up to 9 years after infection [19]. Moreover, through brain volume measurements using magnetic resonance imaging, researchers found that several ICU sepsis survivors had a significant decline in cognitive ability, which was related to left hippocampal atrophy, and they speculated that this lesion was irreversible [20].

According to the findings of the current study, at 24, 48, and 72 h after admission, the serum levels of S-100β, GFAP, and MDA in the SAE group were significantly increased, while the SOD level was considerably less than that of the control group. Furthermore, the serum levels of S-100β, GFAP, and MDA showed significant fluctuations at 72 h, possibly related to physiological changes in the body. In addition, qPCR analysis of isolated PBMCs revealed that the transcription levels of *PEBP-1*, *15-LOX*, and *ACSL4* in the peripheral blood were significantly greater in the SAE group at 24, 48, and 72 h after admission compared to the control group, while the transcription levels of *GPX4* and *SLC7A11* were significantly less. Meanwhile, the transcription levels of *PEBP-1*, *GPX4*, *SLC7A11*, and *ACSL4* showed significant fluctuations at 72 h, suggesting a relationship between the physiological process of SAE and lipid peroxidation ferroptosis. Moreover, tests at different time points were conducted to investigate the dynamic changes in the occurrence and course of the disease, which can help to select the appropriate timing for treatment and intervention. According to the results, significant fluctuations were observed in the serum levels of S-100β, GFAP, and MDA in the patients in the SAE group within 72 h after admission. Similarly, qPCR analysis of PBMCs showed significant fluctuations in the transcription levels of *PEBP1*, *GPX4*, *SLC7A11*, and *ACSL4* within 72 h. Further research should explore the reasons behind these fluctuations and their relationship with patient prognosis. In this study, the expression of S-100β and NSE varied between humans and animals, while some other markers such as SOD, MDA, and GPX4 showed similar trends. These differences may be attributed to species specificity and physiological variations. Furthermore, these differences provide some insights into the feasibility and reliability of translating the research findings into clinical applications.

In this study, the neurobehavioral scores of the mouse model were compared, the morphology of hippocampal cells was observed under a microscope after H&E staining, the changes in brain damage biomarkers (e.g., S-100β and GFAP) were investigated, and these findings were validated with clinical patient samples. The results confirmed that the mice developed hippocampal injury after CLP surgery. Additionally, the ferrous ion content in the damaged hippocampus significantly increased. Following therapy with the iron chelating drug DFO, the damage and accumulation of ferrous ions were improved, suggesting that ferroptosis may play a role in the brain damage mechanism of SAE.

Ferroptosis is characterized by disrupted intracellular iron ion metabolism, increased oxygen free radicals, and cell membrane destruction due to lipid peroxidation, resulting in programmed cell death [21]. Lipid peroxidation occurs when hydrogen atoms are lost from lipids due to free radicals or enzymes, resulting in oxidation and damage to the fatty carbon chains, generating oxygen free radicals, lipid hydroperoxides, and reactive aldehydes (e.g., MDA and 4-HNE), which are cytotoxic substances ultimately leading to cell death [22]. Polyunsaturated fatty acids (PUFAs), phosphatidylethanolamines (PEs), and degraded membrane components play important roles in this process. Oxygen free radicals extract hydrogen atoms from PUFAs, generating lipid radicals, which then combine with oxygen molecules to form lipid peroxide radicals (LOO·) [23]. LOO· extracts hydrogen atoms from PUFAs, generating lipid hydroperoxides (LOOH) and participating in the initiation of ferroptosis. GPX4 is a core regulatory gene in ferroptosis. It binds with the cofactor reduced glutathione to reduce LOOH back to their corresponding lipid alcohols, thus maintaining lipid homeostasis, preventing excessive accumulation of oxygen free radicals, and playing a crucial role in combating lipid peroxidation [24]. When GPX4 activity is blocked, it fails to repair the persistent generation of LOOH, triggering ferroptosis. Ferroptosis has been found to be closely related to neurological disorders [25], which are associated with the levels of PUFA and other fatty acids in the brain. Under certain pathological conditions, it can become a target of lipid peroxidation [26]. Therefore, iron chelators have been applied to improve the clinical symptoms of neurodegenerative diseases [27].

The current study revealed that the mitochondrial cristae in the hippocampus of the SAE mice were significantly reduced at 24 h after surgery, exhibiting morphological characteristics of ferroptosis such as an increased membrane density, partial vacuolar degeneration, and outer membrane rupture. Additionally, the expression of active aldehydes (e.g., MDA and 4-HNE) in the hippocampal tissues of the SAE group was significantly upregulated, while GPX4 was downregulated, indicating that hippocampal injury in SAE mice is associated with lipid peroxidation. After treatment with the iron-chelating agent DFO, the expression of the aforementioned active aldehydes was downregulated, demonstrating that iron chelation can alleviate lipid peroxidation in the hippocampus of SAE mice. However, the regulatory mechanism underlying lipid peroxidation-triggered ferroptosis and hippocampal injury in SAE mice is still not fully understood.

Research on the relationship between SAE and ferroptosis is still limited. Some studies have shown that ferroptosis is associated with hippocampal neuron damage, and DFO can activate the nuclear factor erythroid 2-related factor 2/GPX4 signaling pathway, thereby alleviating hippocampal injury and cognitive impairment in SAE mice [28]. The western blot results of this study showed that compared to the sham group, the SAE group exhibited upregulation of PEBP-1 and 15-LOX expression, while GPX4 expression was downregulated. These findings suggest that hippocampal injury in SAE mice may be connected to lipid peroxidation resulting from the suppression of GPX4 mediated by the PEBP-1/15-LOX complex. Phospholipid lysophosphatidylcholine acyltransferase 3 and ACSL4 are involved in the generation of arachidonic acid-phosphatidylethanolamine (AA-PE) [29, 30]. Subsequently, AA-PE is oxidized by 15-LOX to form lipid peroxides, triggering ferroptosis. 15-LOX and PEBP-1 form a complex anchored to the cell membrane, further generating 15-hydroxyeicosatetraenoic acid (15-HETE)-PE. 15-HETE-PE can inhibit the activity of GPX4, which is an important signal of ferroptosis [31]. The regulation of ferroptosis by ferrostatin-1 is achieved by inhibiting the PEBP-1/15-LOX complex rather than directly inhibiting 15-LOX [32]. In recent years, the PEBP-1/15-LOX complex also has been studied as a therapeutic target for several diseases. Vitamin E can reduce the expression of 15-LOX in the hippocampus, inhibit the accumulation of MDA and iron, and reduce seizures and neuronal damage [33]. Based on the current research, the pathogenesis of SAE is related to the PEBP-1/15-LOX/GPX4 pathway. SAE is divided into acute and chronic stages, with chronic patients exhibiting persistent neurocognitive impairment and brain damage. This study primarily focused on early neuronal damage in SAE. In the chronic stage of SAE, additional research is required to determine the precise mechanisms underlying cognitive dysfunction and neuronal damage.

### Study strengths and limitations

The strengths of this study are that it combines clinical data and animal models, providing comprehensive observations and experiments on pediatric patients and mice. This approach enhances the credibility and generalizability of the research findings. This study also identified potential therapeutic targets for treating neuronal injury in SAE and offers valuable insights into the potential mechanisms of ferroptosis in neurological disorders. Although this study provided evidence of the involvement of lipid peroxidation-induced ferroptosis in SAE, the specific mechanisms were not directly elucidated. Therefore, further research is needed to uncover the detailed mechanisms underlying this relationship.

## Conclusions

This study demonstrated that PEBP-1/15-LOX-mediated lipid peroxidation is the initial factor triggering ferroptosis. This pathway is associated with the pathogenesis of SAE, and the inhibitor DFO can inhibit the PEBP-1/15-LOX pathway, restore GPX4 activity, and improve SAE-induced hippocampal injury. This study primarily focused on early neuronal damage in SAE. Future research can extend the time window to further elucidate the relationship between the PEBP-1/15-LOX complex and chronic cognitive impairment induced by SAE as well as to explore new clinical treatment methods targeting this pathway. The clinical relevance of this study lies in revealing the relationship between neuronal injury and lipid peroxidation-induced ferroptosis in SAE. These findings provide potential therapeutic targets for the management of neuronal injury in SAE patients and valuable insights into the potential mechanisms of ferroptosis in neurological disorders.

### Supplementary Information

**Supplementary Material 1. **

## Data Availability

All data generated or analyzed during this study are included in this published article.

## Abbreviations

SAE: Sepsis-associated encephalopathy
CLP: Cecal ligation and puncture
PEBP-1: Phosphatidylethanolamine-binding protein 1
15-LOX: 15-Lysyl oxidase
GPX4: Glutathione peroxidase 4
DFO: Deferoxamine
PBMCs: Peripheral blood mononuclear cells
GFAP: Glial fibrillary acidic protein
SOD: Superoxide dismutase
MDA: Malondialdehyde
ELISAs: Enzyme-linked immunosorbent assays
H&E: Hematoxylin and eosin
TEM: Transmission electron microscopy
BCA: Bicinchoninic acid
PVDF: Polyvinylidene fluoride
NSE: Neuron-specific enolase
4-HNE: 4-Hydroxynonenal
IL-6: Interleukin-6
TNF-α: Tumor necrosis factor alpha
OD: Optical density
AA-PE: Arachidonic acid-phosphatidylethanolamine
