# Supplementary material for: Lipid peroxidation-induced ferroptosis as a therapeutic target for mitigating neuronal injury and inflammation in sepsis-associated encephalopathy: insights into the hippocampal PEBP-1/15-LOX/GPX4 pathway
Source: Lipids Health Dis. 2024 Apr 29;23:128. doi: 10.1186/s12944-024-02116-x (PMC11057122; doi:10.1186/s12944-024-02116-x)
Supplement: Supplementary file 1 — Supplementary Material 1. [file 12944_2024_2116_MOESM1_ESM.docx]

**Supplementary Table 1. Basic information of the clinical patients**

| Group | Index | Sex | Age  years | WBC  10^9/L | CRP  mg/L | ALT  U/L | AST  U/L | Creatinine  μmol/L | Urea  mmol/L | GCS | Infection site | Pathogen |
| --- | --- | --- | --- | --- | --- | --- | --- | --- | --- | --- | --- | --- |
| 1 | 1 | 0 | 1.1 | 14.88 | 116.91 | 37 | 58 | 26 | 5.26 | 8 | 2 | 1 |
| 1 | 2 | 0 | 11.9 | 18.64 | 31.27 | 72 | 115 | 33 | 8.19 | 7 | 0 | 0 |
| 1 | 3 | 1 | 55 | 36.1 | 18.81 | 114 | 36 | 30 | 6.11 | 9 | 3 | 4 |
| 1 | 4 | 0 | 2.5 | 11.83 | 63.21 | 48 | 100 | 21 | 3.61 | 6 | 0 | 0 |
| 1 | 5 | 1 | 16 | 9.7 | 61.21 | 25 | 67 | 29 | 4.52 | 5 | 1 | 1 |
| 1 | 6 | 0 | 3.3 | 11.59 | 110.76 | 69 | 45 | 17 | 2.49 | 7 | 0 | 0 |
| 1 | 7 | 1 | 16 | 11.66 | 1.76 | 58 | 99 | 22 | 4.4 | 8 | 0 | 3 |
| 1 | 8 | 0 | 72 | 2.49 | 19.64 | 59 | 99 | 26 | 3.25 | 5 | 2 | 2 |
| 1 | 9 | 0 | 45 | 14.39 | 33.74 | 27 | 67 | 22 | 5.31 | 7 | 0 | 0 |
| 1 | 10 | 0 | 1.13 | 2.07 | 143.9 | 55 | 40 | 23 | 4.26 | 6 | 4 | 2 |
| 2 | 1 | 0 | 17 | 14.95 | 169.55 | 19 | 36 | 26 | 3 | 10 | 0 | 0 |
| 2 | 2 | 1 | 2.3 | 8.14 | 7.97 | 13 | 39 | 23 | 1.13 | 12 | 0 | 0 |
| 2 | 3 | 1 | 18 | 7.27 | 2.79 | 41 | 53 | 59 | 3.35 | 13 | 0 | 0 |
| 2 | 4 | 0 | 22 | 4.8 | 2.12 | 22 | 31 | 30 | 4.42 | 13 | 1 | 2 |
| 2 | 5 | 0 | 26 | 19.69 | 189.87 | 34 | 34 | 15 | 1.75 | 12 | 0 | 0 |
| 2 | 6 | 0 | 16 | 2.71 | 0.9 | 22 | 36 | 46 | 4.71 | 10 | 0 | 0 |
| 2 | 7 | 1 | 2.1 | 1.49 | 1.42 | 172 | 166 | 36 | 11.96 | 11 | 0 | 3 |
| 2 | 8 | 0 | 2.5 | 17.64 | 0.95 | 30 | 44 | 25 | 2.76 | 11 | 0 | 0 |
| 2 | 9 | 0 | 3.7 | 17.13 | 79.74 | 13 | 66 | 19 | 3.64 | 12 | 5 | 1 |
| 2 | 10 | 1 | 30 | 19.64 | 41.55 | 25 | 128 | 23 | 5.9 | 10 | 0 | 0 |

1=SAE; 2=non-SAE 0=male; 1=female

Pathogen: 1. *Escherichia coli*; 2. *Streptococcus pneumoniae*; 3. *Staphylococcus aureus*; 4. *Enterococcus faecalis*; 5. *Haemophilus influenzae*.

Infection site: 1. Lower respiratory tract infection; 2. Urinary tract infection; 3. Gastrointestinal tract infection; 4. Skin and soft tissue infection.
